# Investigation through Animal–Computer Interaction: A Proof-of-Concept Study for the Behavioural Experimentation of Colour Vision in Zoo-Housed Primates

**DOI:** 10.3390/ani14131979

**Published:** 2024-07-04

**Authors:** Vinícius Donisete Lima Rodrigues Goulart, Robert John Young

**Affiliations:** 1 Transportation Research and Environmental Modelling Laboratory—TREM, Institute of Geosciences, Universidade Federal de Minas Gerais, Belo Horizonte 31270-901, Brazil; viniciusdonisete@gmail.com; 2 School of Science, Engineering and Environment, Peel Building, University of Salford Manchester, Salford M5 4WT, UK

**Keywords:** colour vision, zoo, animal experimentation, behavioural research, sensory ecology, animal–computer interaction, ACI, animal welfare

## Abstract

**Simple Summary:**

Animal–computer interactions provide an opportunity for behavioural investigations. Once appropriate interfaces are established, it is possible to provide new possibilities for improving animal welfare and experimentation. Zoos are an important repository of animals and provide a great source of biological knowledge. By using accessible materials and with low levels of programming, we were able to develop a safe and reliable design for testing the sensory abilities of New World primates. The proof-of-concept for testing colour vision through a behavioural experiment resulted in engagement from the tested animals and an alternative for investigating the sensory abilities of this complex group of animals. In conclusion, we encourage the use of animal-computer interaction frameworks to enrich and develop scientific knowledge from captive animals.

**Abstract:**

Zoos are an important repository of animals, which have a wide range of visual systems, providing excellent opportunities to investigate many comparative questions in sensory ecology. However, behavioural testing must be carried out in an animal welfare-friendly manner, which is practical for zoo staff. Here, we present a proof-of-concept study to facilitate behavioural research on the sensory ecology of captive primates. A system consisting of a tablet computer and an automated feeder connected wirelessly was developed and presented to captive primate species to evaluate interactions with and without previous training. A colour stimulus, analogous to the Ishihara test, was used to check the level of interaction with the device, supporting future studies on sensory ecology with zoo animals. Animals were able to use the system successfully and displayed signs of learning to discriminate between the visual stimuli presented. We identified no risk for small primates in their interactions with the experimental setup without the presence of keepers. The use of electronic devices should be approached with caution to prevent accidents, as a standard practice for environmental enrichment for larger animals (e.g., spider monkeys). In the long term, the system developed here will allow us to address complex comparative questions about the functions of different visual systems in captive animals (i.e., dichromatic, trichromatic, etc.).

## 1. Introduction

Zoos are an important repository of animals, housing tens of thousands of different animal species around the world and millions of individuals, providing environmental education, aiding conservation efforts, and contributing to scientific research [1,2,3]. The use of artificial elements in exhibits has received some criticism from advocates of strictly naturalistic exhibits advocates, which may have caused reticence in the uptake of modern technology in research [4]. Zoos have a great potential for behavioural research, but for pure research, such as sensory ecology, this resource is largely under-utilised. The most obvious explanation for this is that zoos focus on conservation research, along with the fact that zoos have many other restrictions on the type of research they will allow [5]. For instance, a challenge faced by researchers conducting studies in zoos is the restriction on handling animals or modifying the enclosures, which is often necessary to achieve the best experimental design [6]. Despite the possible mischaracterization of naturalistic enclosures, environmental enrichment devices such as touchscreen computers have not affected the zoo visitors’ perceptions towards animal welfare [7]. Zoo-based research often faces difficulties with small sample sizes, which can be overcome by multi-zoo studies with equipment that is easy to adapt to different enclosures. Thus, using methods less demanding to keepers’ routines by automating data collection and animal–computer interaction experiments shared among zoological institutions could be the answer. Furthermore, zoo-based research should not have a negative impact on animal welfare. Given that these restrictions (i.e., animal welfare and keeper time) are red lines that zoos will not cross, researchers need to develop alternative methods for conducting their research.

Computer interfaces are centred in human abilities, such as the visual system and physical input hardware (i.e., keyboard or mouse); however, some interfaces are cross-species, such as audio, video tracking, accelerometers, and haptic sensors [8]. This leads to the development of interfaces appropriate for animal use. The animal–computer interaction (ACI) is a growing field in environmental enrichment and the animal experimentation field [9]. It can be a source of stimuli in animal enclosures, providing an enriched environment for better animal welfare [10]. Moreover, this approach can be used to experiment and test hypotheses, thereby allowing the study of animal behaviour in animal collections such as zoos [11].

Tablet computers are an affordable technology to investigate the sensory abilities of different animals: their thin profile and touch interface allow an intuitive interaction when compared to traditional computers, where mouse and keyboards comprise the main input hardware. Therefore, there is no need to translate physical movement into virtual movement [9]. The number of sensors present in commercially available tablets increases the possibility of their application to address behavioural and sensory questions. For instance, accelerometers built in the device can be used to record positional behaviour [12]; proximity sensors can trigger data loggers [13]; or the display and touch screen can be used for stimuli presentation [14,15].

One challenge imposed by using touch screens for colour stimuli presentation is that colour replication accuracy cannot be guaranteed. For instance, sensory ecology and vision research usually employs no digital compression in photos taken from cameras, colour checker cards, or colour-referenced stimuli [16,17]. Therefore, any behavioural research should take into consideration variations in colour stimuli presented on different screen types and proceed with calibration methods.

Colour discrimination in animals is related to the number of photoreceptors sensitive to different wavelengths of light [18]. Vertebrates, such as the nocturnal owl monkey (*Aotus* sp.), have one photoreceptor and are not able to distinguish colours (monochromats); most mammals that have two photoreceptors are dichromats; the majority of Old World primates are trichromats and have three photoreceptors; fishes, reptiles, and birds are tetrachromats and have the best colour vision acuity among vertebrates [19,20]. The greatest number of photoreceptors has been found in the mantis shrimp (*Neogonodactylus oerstedii*), which has 12 photoreceptors [21]. Therefore, when using an animal–computer interface display to perform behavioural research, it should be appropriate to the colour vision system of the species in terms of the colour discrimination tasks involved.

New World primates have a polymorphic colour vision system [22,23]. Within the same species, males are obligatory colour blind with a colour vision similar to a red–green colour blind human, whereas females can be either dichromats or trichromats [24,25,26]. Each phenotype has its own advantages: dichromats are best suited for vision in low light levels and camouflage breaking, whereas trichromats outperform dichromats in detecting ripe food sources or detecting predators in photopic light levels [27,28,29,30,31]. For instance, dichromat phenotypes under natural environmental conditions are notably more efficient at detecting camouflaged insects, especially in low light, suggesting an ability to break camouflage for detecting food sources and predators [32]. The trichromacy advantage conferred for ripe fruits foraging in New World primates is supported by field studies on intake rates of conspicuous coloured fruits favouring an trichromatic phenotype advantage conferring nutritional benefit on fruit foraging [33]. Further studies investigating social cooperative behaviour related to visual perception would help us understand the role of polymorphic colour vision in New World primates.

Here, we present a proof-of-concept study concerning the realisation of behavioural vision research in zoos. Thus, we investigated the use of commercial devices (i.e., off-the-shelf tablet computers) and the development of a customisable feeder to be used by keepers in zoo enclosures without causing disturbances to animal management or animal welfare.

## 2. Materials and Methods

### 2.1. Animals

A mixed group of two marmoset individuals (*Callithrix geoffroyi*), one male and one female, and three titi monkeys (*Plecturocebus cupreus*), a pair and a young male, kept in the same enclosure in Twycross Zoo, United Kingdom, were subjected to experimental sessions. The experimental sessions took place in the same enclosure where the animals were housed; the animals were free to move outdoors. There was no need for handling or capturing the animals during the study, and, therefore, this did not occur.

A single group of three variegated spider monkeys (*Ateles hybritus*), two females and one male, also housed in Twycross Zoo, were also used in the experiment. The experimental setup was placed in an animal management area, with the animals being free to move to their normal indoor enclosure or outdoors during the experimental session.

The animals were not deprived of food, and routine feeding and food enrichments were maintained during this study. The experimental sessions were performed according to the keeper’s routine husbandry sessions from December 2016 to April 2017. Each session lasted 10 min and would have been terminated in case of any undesired circumstances (e.g., aggression) towards the apparatus or group members.

### 2.2. Stimuli

**Figure 1:**
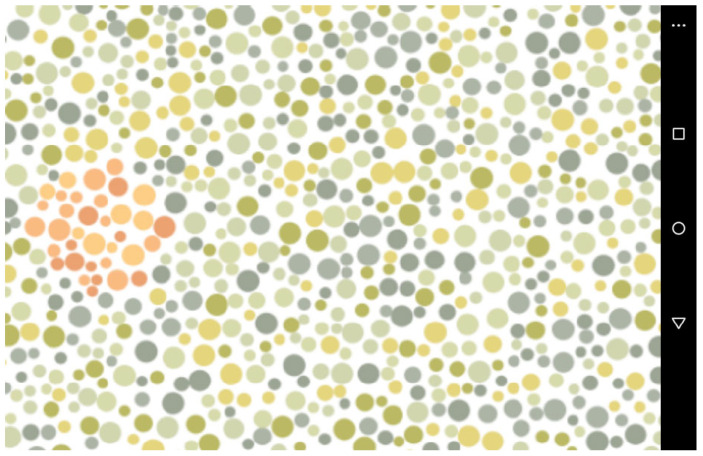
A screen capture of the colour stimuli created to investigate the interaction of captive primates with the apparatus developed to behaviourally investigate colour vision. A food reward was provided by a feeder connected via Bluetooth when the red dots were pressed. A horn was played by the tablet when the green dots were touched.

The presented stimuli comprised an image composed of circles in varying sizes and colours, comparable to the Ishihara colour blind test (i.e., pseudoisochromatic plate), which was produced using Java code in Processing v2.2.1. Each circle had a maximum diameter of 22 pixels and a minimum diameter of 8 pixels. A white background was set, and the code produced a random pattern where a static figure in PNG file format was placed randomly on a canvas of 1024 × 576 pixels. The PNG file had no background, and the outline and filling of the image were replaced by red-coloured circles. A rounded shape was used to produce a red target (Figure 1).

An Android app was created using the MIT app inventor, where the images created were shown on a tablet screen (Amazon Fire). If the target was touched, a clicker sound was emitted, and the target’s position changed. If the wrong area was touched, a horn sound was played, and the target did not change its location. The tablet had a wireless connection to a feeder, which provided a raisin as a food reward if the right area in the tablet’s screen was touched. A red–green colour blind human volunteer checked whether the target was visible or not. Also, a spectrophotometer, Ocean Optics USB 2000+ VIS-NIR (Halma plc, Amersham, UK), attached to a light source (LS-1 Tungsten Halogen light, Ocean Optics, Halma plc, Amersham, UK), was used to collect the relative irradiance from the tablet screen, and the colours of the target and background were compared by calculating their JND (Just Noticeable Difference) modelling trichromatic and dichromatic phenotypes. The tablet used did not have readily available colour calibration; therefore, the colours selected for developing the pseudoisochromatic image used in this study were calibrated by programming, selecting adequate hex-codes given the validation by volunteers and spectrophotometer measurements. Sensory analysis was performed using R and the package Pavo [34,35].

### 2.3. Apparatus

**Figure 2:**
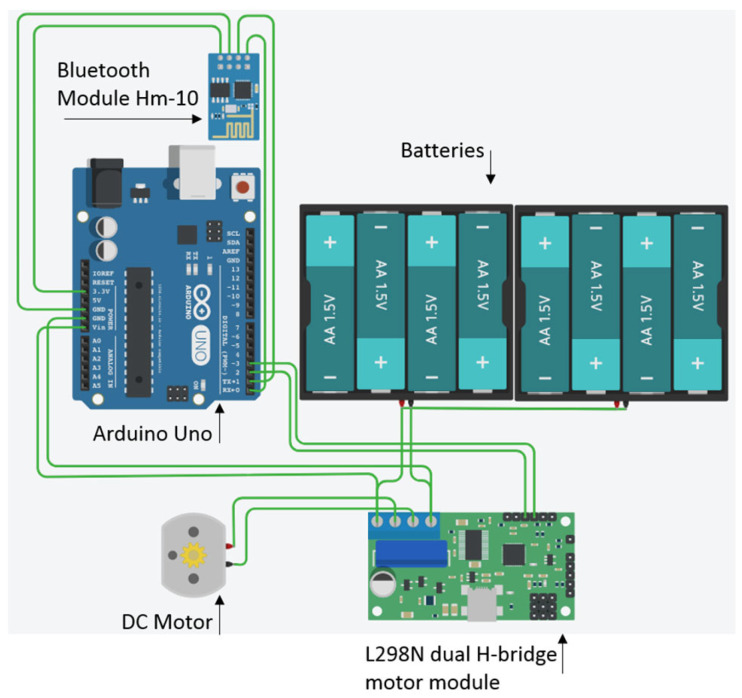
A schematic diagram of the automated wireless feeder used in behavioural colour vision research.

**Figure 3:**
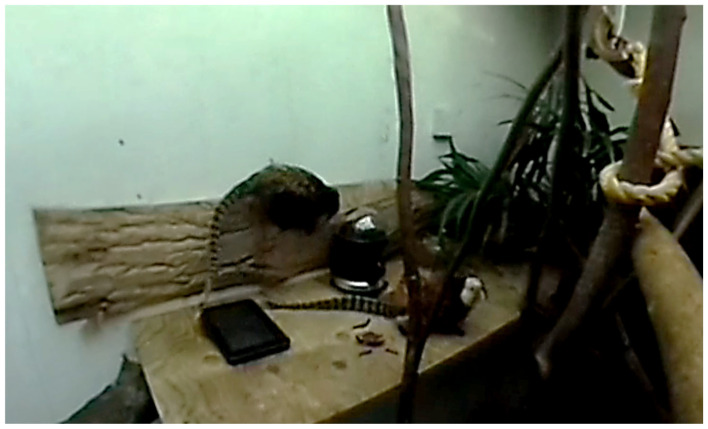
Experimental apparatus during the performance of behavioural tests.

The feeder consisted of a DC motor attached to a plastic spiral that turned, pushing the reward (raisins) from a plastic container. An Arduino UNO microcontroller board (ATmega328P) was connected to the DC motor using an L298N dual H-bridge DC motor driver module. An Hm-10 Bluetooth module was also connected to the Arduino, allowing the wireless connection to the tablet. A Kindle Fire tablet with a 7-inch screen with a resolution of 1024 × 600 pixels and a rugged case was used (Table 1; Figure 2 and Figure 3). A Ricoh theta 360 degrees camera in a protective case was used to film the experimental sessions.

### 2.4. Procedure

The feeder, tablet, and camera were placed inside the marmoset/titis enclosure for 10 trials. At the start of the session, a raisin was placed on top of the correct stimuli to help the association between the red target and the reward. No training was performed with the marmosets and titis.

In the spider monkey enclosure, the camera and the feeder were positioned outside the enclosure, and the keeper held the tablet, allowing the animals to touch the screen through the mesh. The training consisted of placing the raisins on top of the target during the first two minutes of the presentation, leaving the animals free to interact with the tablet after the presentation. Behavioural data were collected ad libitum, reporting behaviours expressed during interactions with the device. The time and the result of the interaction were recorded. A total of 11 trials were performed.

The data were checked for normality, and non-parametric tests were used as we found the data did not fulfil parametric requirements. A logistic regression was used to evaluate the association between the tablet and the feeder. The number of sequential interactions between the tablet and feeder occurred during the experimental session in the marmoset/titi enclosure. The frequency of training (only with the spider monkey group) and the number of correct uses was checked for correlation to the accumulative time using a Spearman’s rank correlation test. All statistics were performed in the R statistical computing language [35].

## 3. Results

### 3.1. Stimuli

**Figure 4:**
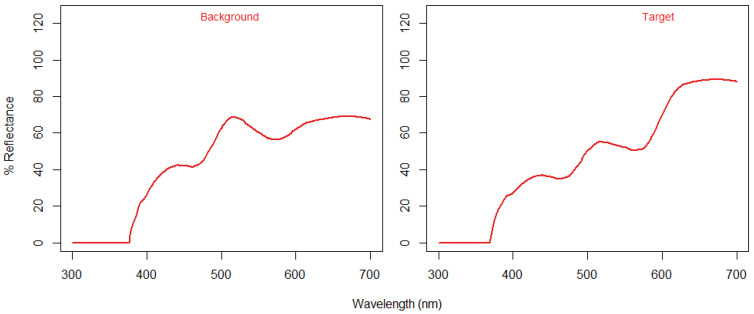
The relative irradiance of the pseudoisochromatic image taken from a commercial tablet (Kindle Fire).

The colours suitable for a colour blind test are shown in Table 2 and Figure 4. By averaging the target colours and background colour from the tablet screen using their relative irradiance values, we found a colour distance of 2.8040 JND (Just Noticeable Difference) for a trichromatic phenotype, and 0.2849 JND for a dichromat viewer. Therefore, the target and background were not distinguishable in colour for dichromat phenotypes.

### 3.2. Marmosets and Titis

A total of 82.52 min of stimuli presentation were obtained from ten experimental sessions. The device screen was sensitive to the touch of marmosets and titis (activated also by accidental touches, such as inspections and stepping on it). Interactions with the device lasted an average of 16 s with a range of 2 to 101 s. During the experimental session, device interactions accounted for 24.92% of the total time. Most of the interactions in the mixed species enclosure were performed by the marmosets (92.21%), who expelled the titis from the testing platform. We observed an association between the tablet and the feeder, with the animals inspecting the tablet and the feeder sequentially and looking to the display. From the experimental sessions, we did not find marmosets and titis using the device only to receive the rewards but also as an environmental enrichment as they were curious about activating the feeder. Likewise, the individuals associated the tablet with the feeder significantly, according to a logistic regression (β ± SE = 0.001 ± 0.0003; Z = 4.131; *p* < 0.001).

### 3.3. Spider Monkeys

A total of 11 experimental sessions were performed, with a total interaction time of 66.68 min. All time was used by the experimental animals interacting with the device due to a different experimental setup from titis and marmosets. We found that spider monkeys were able to learn to use the device because they were able to receive rewards from the feeder five times in a row (i.e., no incorrect interactions). This result suggests that the female individual may possess trichromatic colour vision, indicating behavioural experiments as a potential method for colour vision assessment in zoo animals. Agonistic behaviours were observed in two experimental sessions, where the male, not being able to select the right colour target, repelled the female from receiving the reward. No collaborative behaviour was observed between the males and females. The number of times that the keeper had to demonstrate and habituate the animal to use the device (i.e., training) was negatively correlated with the cumulative time of the experiment (rs = −0.6245; N = 11; *p* = 0.0399). However, we found no correlation between the cumulative time and correct use of the device (rs = −0.2535, N = 11; *p* = 0.4520). Overall, the experimental animals were participative and appeared motivated to use the experimental apparatus in the behavioural tests.

## 4. Discussion

The zoo staff found the device simple to use; it was easy to build and met the requirements for behavioural vision research in zoos. The touch screen of the commercial tablet with a rugged case and screen protector was reactive to the primates’ touch. Even small primates (i.e., <500 g), such as marmosets, were able to interact and receive a reward. Therefore, the device is a suitable alternative to expensive scientific equipment used in some behavioural research [15]. Efforts to bridge the gap between captive and wild animal research will provide more research opportunities and support an interdisciplinary approach [36]. For instance, research on colour vision requires the knowledge of the physical properties of light, physiological aspects of colour vision, and implications of different phenotypes on the sensory ecology [37,38,39]. Methodologies that allow us to access the sensory abilities of animal subjects are crucial to increasing knowledge in the field.

Two major hypotheses about the importance of polymorphic colour vision for New World primates are niche divergence and mutual benefit association [40,41]. By applying similar setups, the niche divergence could be verified by changing the stimuli type from colour conspicuous to cryptic and analysing the behaviour demonstrated by the different phenotypes. The mutual benefit association hypothesis could be tested by investigating collaboration among individuals in response to a given colour vision task. The use of animal–computer interaction-based experiments with captive primates contributes substantially to the collection of behavioural data and controlling environmental variables.

The marmosets used in this study also inhabit forest fragments in cities, are frequently in contact with city dwellers, and are often hand-fed [42,43,44]; yet, they preserve many aspects of their natural behaviour [42]. The small sample sizes that often limit the value of research in zoos could be reduced by investigating urban animals. The portability of the setup used in this proof-of-concept study could easily be extrapolated to research in urban environments, which are more flexible regarding the access to animals and possibilities of modifying the environment. The marmosets in our study were able to associate the feeder and the tablet without assistance (training) from keepers. Thus, further studies with urban marmosets should be investigated.

One of our main goals with this study was to verify the use of zoo enclosures as an experimental area, having the keepers performing the experiment. Keepers have close contact with their animals, and their interaction is relevant in designing scientific experiments [45]. It was possible to shape the behaviour of spider monkeys with a few presentations of the experimental equipment. Nevertheless, preparation to perform the research and interest from the zoo staff are critical for success. Fortunately, Twycross Zoo is an institution interested in supporting behavioural research. Further presentations could be performed without the keeper’s presence, as the experimental setup permits its use without any human assistance.

As the opportunity to manipulate zoo enclosures is limited, certain precautions must be taken to ensure the successful realisation of experiments. We found that mixed species enclosures can lead to certain species not being able to participate in the experiments. This should be considered in future studies concerning socially housed species. A critical aspect of video recording is illumination. Indoor areas should be assessed regarding their light sources, since the identification of the individual performing the behaviour is important in behavioural studies. Keepers often have a profound knowledge of each individual animal in their care, being able to recognise them without marking them. However, individual animal identification might be not possible if a ‘control’ researcher is used to analyse the recordings, for instance, if a double-blind experimental design is used, where the executive and analysis of experiments are performed by two different researchers independently.

Touch-sensitive tablet computer screens can have different colour reproduction from the colour selected on the computer used for programming the tablet. However, we controlled for this by selecting colours that were not visible to a dichromatic (human) colour vision phenotype. This problem is of major concern when developing colour-based tasks in which the colour stimuli may be altered on different screens. Despite this, it was possible to use a commercial tablet to generate colour targets and backgrounds that are not detectable by red–green colour blind viewers.

Unfortunately, due to time limitations, it was not possible to proceed with detailed behavioural studies of vision, but the concept proved that a computer tablet-based system can be used to behaviourally assess visual perception in zoo-housed primates.

## 5. Conclusions

The experimental design focused on the feasibility of animal–computer interaction-based experiments with captive animals to study sensory behaviour, which is expected to vary among the experimental subjects. We successfully tested a safe device that provided an interactive interface for studying animal perception with little impact on keepers’ routines. We observed learning in spider monkeys with minimal training, and marmosets showed a significant interest in interacting with the system. Further studies can evaluate the differences in interactions between group members and infer about perception abilities.

The interest shown for interactive devices, such as touch screens and reward mechanisms, has the potential to be used as environmental enrichment in small primate enclosures. The use of simple builds with components “off-the-shelf” can be employed securely for small primate species. No aggressive behaviours, which could terminate the experimental sessions, were observed, and no damaging behaviours (e.g., biting) were observed towards the device.

Behavioural research involving the development of interfaces for interactive devices can contribute to increasing the scientific knowledge of species biology, conciliating the demands from animal management in zoological institutions, and providing the controlled environment needed for experimentation.

## Figures and Tables

**Table 1:** The components used to build a visual stimuli presentation device to behaviourally measure colour vision.

Component	Cost
Kindle Fire tablet computer, 7-inch 1024 × 600 screen, 313 g	GBP 35.00
Arduino Uno microcontroller board ATmega328P	GBP 17.30
DC motor-powered dispenser	GBP 5.00
Bluetooth module Hm-10	GBP 6.00
L298N dual H-bridge DC motor module	GBP 5.00
Rugged tablet case with screen protector	GBP 15.00

**Table 2:** Colour references of the pseudoisochromatic stimuli found to be undistinguishable by di-chromatic colour vision phenotype (i.e., a red–green colour blind individual).

Colour Category	Hex-Colour Code	CIE-Lab L	CIE-Lab A	CIE-Lab L
Green	d9CA594	66.6087	−6.3958	7.7143
Green	ACB4A5	72.3152	−5.5814	6.6789
Green	BBB946	73.3536	−13.7646	56.6906
Green	D1D6AF	84.3514	−8.2588	18.7266
Green	D7DAAA	85.7519	−8.8320	23.3500
Green	E5D57D	84.8571	−6.2664	45.3062
Red	EBA170	72.4431	22.2198	36.4731
Red	F9BB82	80.3436	15.6987	37.4752
Red	FCCD84	85.0087	7.6967	42.4568

## Data Availability

The data are available upon request from the author.

## References

[1] Barongi R., Fisken F.A., Parker M., Gusset M. (2015). Committing to Conservation: The World Zoo and Aquarium Conservation Strategy.

[2] Patrick P.G., Matthews C.E., Ayers D.F., Tunnicliffe S.D. (2007). Conservation and Education: Prominent Themes in Zoo Mission Statements. J. Environ. Educ..

[3] Tribe A., Booth R. (2003). Assessing the Role of Zoos in Wildlife Conservation. Hum. Dimens. Wildl..

[4] Fernandez E.J., Martin A.L. (2021). Animal Training, Environmental Enrichment, and Animal Welfare: A History of Behavior Analysis in Zoos. J. Zool. Bot. Gard..

[5] Kleiman D.G. (1992). Behavior Research in Zoos: Past, Present, and Future. Zoo Biol..

[6] Hosey G.R. (1997). Behavioural Research in Zoos: Academic Perspectives. Appl. Anim. Behav. Sci..

[7] Jacobson S.L., Hopper L.M., Shender M.A., Ross S.R., Leahy M., McNernie J. (2017). Zoo Visitors’ Perceptions of Chimpanzee Welfare Are Not Affected by the Provision of Artificial Environmental Enrichment Devices in a Naturalistic Exhibit. J. Zoo Aquar. Res..

[8] McGrath R.E. (2019). Species-Appropriate Computer Mediated Interaction. Proceedings of the CHI ’09 Extended Abstracts on Human Factors in Computing Systems.

[9] Ritvo S.E., Allison R.S. (2017). Designing for the Exceptional User: Nonhuman Animal-Computer Interaction (ACI). Comput. Hum. Behav..

[10] Wirman H., Zamansky A. (2016). Toward Characterization of Playful ACI. Interactions.

[11] Mancini C. (2011). Animal-Computer Interaction: A Manifesto. Interactions.

[12] Graf P.M., Wilson R.P., Qasem L., Hackländer K., Rosell F. (2015). The Use of Acceleration to Code for Animal Behaviours; A Case Study in Free-Ranging Eurasian Beavers Castor Fiber. PLoS ONE.

[13] Nathan R., Getz W.M., Revilla E., Holyoak M., Kadmon R., Saltz D., Smouse P.E. (2008). A Movement Ecology Paradigm for Unifying Organismal Movement Research. Proc. Natl. Acad. Sci. USA.

[14] Takemoto A., Miwa M., Koba R., Yamaguchi C., Suzuki H., Nakamura K. (2015). Individual Variability in Visual Discrimination and Reversal Learning Performance in Common Marmosets. Neurosci. Res..

[15] Takemoto A., Izumi A., Miwa M., Nakamura K. (2011). Development of a Compact and General-Purpose Experimental Apparatus with a Touch-Sensitive Screen for Use in Evaluating Cognitive Functions in Common Marmosets. J. Neurosci. Methods.

[16] Melin A.D., Kline D.W., Hickey C.M., Fedigan L.M. (2013). Food Search through the Eyes of a Monkey: A Functional Substitution Approach for Assessing the Ecology of Primate Color Vision. Vis. Res..

[17] Pessoa D.M.A., Cunha J.F., Tomaz C., Pessoa V.F. (2005). Colour Discrimination in the Black-Tufted-Ear Marmoset (*Callithrix penicillata*): Ecological Implications. Folia Primatol..

[18] Yokoyama S. (2000). Molecular Evolution of Vertebrate Visual Pigments. Prog. Retin. Eye Res..

[19] Bowmaker J.K. (2008). Evolution of Vertebrate Visual Pigments. Vis. Res..

[20] Jacobs G.H. (2009). Evolution of Colour Vision in Mammals. Phil. Trans. R. Soc. B.

[21] Thoen H.H., How M.J., Chiou T.-H., Marshall J. (2014). A Different Form of Color Vision in Mantis Shrimp. Science.

[22] Hunt D.M., Dulai K.S., Cowing J.A., Julliot C., Mollon J.D., Bowmaker J.K., Li W.-H., Hewett-Emmett D. (1998). Molecular Evolution of Trichromacy in Primates. Vis. Res..

[23] Jacobs G.H. (2007). New World Monkeys and Color. Int. J. Primatol..

[24] De Valois R.L., Jacobs G.H. (1968). Primate Color Vision: The Macaque and Squirrel Monkey Differ in Their Color Vision and in the Physiology of Their Visual Systems. Science.

[25] Dulai K.S., Von Dornum M., Mollon J.D., Hunt D.M. (1999). The Evolution of Trichromatic Color Vision by Opsin Gene Duplication in New World and Old World Primates. Genome Res..

[26] Jacobs G.H., Neitz M., Deegan J.F., Neitz J. (1996). Trichromatic Colour Vision in New World Monkeys. Nature.

[27] Dominy N.J., Lucas P.W. (2001). Ecological Importance of Trichromatic Vision to Primates. Nature.

[28] Morgan M.J., Adam A., Mollon J.D. (1992). Dichromats Detect Colour-Camouflaged Objects That Are Not Detected by Trichromats. Proc. R. Soc. Lond. B.

[29] Osorio D., Vorobyev M. (1996). Colour Vision as an Adaptation to Frugivory in Primates. Proc. R. Soc. B..

[30] Pessoa D.M.A., Maia R., De Albuquerque Ajuz R.C., De Moraes P.Z.P.M.R., Spyrides M.H.C., Pessoa V.F. (2014). The Adaptive Value of Primate Color Vision for Predator Detection. Am. J. Primatol..

[31] Verhulst S., Maes F.W. (1998). Scotopic Vision in Colour-Blinds. Vis. Res..

[32] Melin A.D., Fedigan L.M., Hiramatsu C., Sendall C.L., Kawamura S. (2007). Effects of Colour Vision Phenotype on Insect Capture by a Free-Ranging Population of White-Faced Capuchins, *Cebus Capucinus*. Anim. Behav..

[33] Melin A.D., Chiou K.L., Walco E.R., Bergstrom M.L., Kawamura S., Fedigan L.M. (2017). Trichromacy Increases Fruit Intake Rates of Wild Capuchins (*Cebus Capucinus Imitator*). Proc. Natl. Acad. Sci. USA.

[34] Maia R., Eliason C.M., Bitton P., Doucet S.M., Shawkey M.D. (2013). Pavo: An R Package for the Analysis, Visualization and Organization of Spectral Data. Methods Ecol. Evol..

[35] R Core Team (2022). R: A Language and Environment for Statistical Computing.

[36] Shettleworth S.J. (2001). Animal Cognition and Animal Behaviour. Anim. Behav..

[37] Endler J.A. (1993). The Color of Light in Forests and Its Implications. Ecol. Monogr..

[38] Isbell L.A. (2006). Snakes as Agents of Evolutionary Change in Primate Brains. J. Hum. Evol..

[39] Stevens M. (2013). Sensory Ecology, Behaviour, and Evolution.

[40] Melin A.D., Fedigan L.M., Hiramatsu C., Kawamura S. (2008). Polymorphic Color Vision in White-Faced Capuchins (*Cebus Capucinus*): Is There Foraging Niche Divergence among Phenotypes?. Behav. Ecol. Sociobiol..

[41] Hiwatashi T., Okabe Y., Tsutsui T., Hiramatsu C., Melin A.D., Oota H., Schaffner C.M., Aureli F., Fedigan L.M., Innan H. (2010). An Explicit Signature of Balancing Selection for Color-Vision Variation in New World Monkeys. Mol. Biol. Evol..

[42] Duarte M.H.L., Goulart V.D.L.R., Young R.J. (2012). Designing Laboratory Marmoset Housing: What Can We Learn from Urban Marmosets?. Appl. Anim. Behav. Sci..

[43] Goulart V.D.L.R., Teixeira C.P., Young R.J. (2010). Analysis of Callouts Made in Relation to Wild Urban Marmosets (*Callithrix penicillata*) and Their Implications for Urban Species Management. Eur. J. Wildl. Res..

[44] Teixeira B., Hirsch A., Goulart V.D.L.R., Passos L., Teixeira C.P., James P., Young R. (2015). Good Neighbours: Distribution of Black-Tufted Marmoset (*Callithrix penicillata*) in an Urban Environment. Wildl. Res..

[45] Carlstead K. (2009). A Comparative Approach to the Study of Keeper–Animal Relationships in the Zoo. Zoo Biol..

